# Seroprevalance of Crimean–Congo haemorrhagic fever in Bulgarian livestock

**DOI:** 10.1080/13102818.2014.931685

**Published:** 2014-07-08

**Authors:** Robert Barthel, Emad Mohareb, Rasha Younan, Teodora Gladnishka, Nikolay Kalvatchev, Abdel Moemen, Sameh S. Mansour, Cynthia Rossi, Randal Schoepp, Iva Christova

**Affiliations:** ^a^ Naval Medical Center, Division of Infectious Diseases, San Diego, CA, USA; ^b^ Viral and Zoonotic Diseases Research Program, Naval Medical Research Unit #3 (NAMRU-3), Cairo, Egypt; ^c^ Microbiology Department, National Center of Infectious and Parasitic Diseases (NCIPD), Sofia, Bulgaria; ^d^ US Army Research Institute of Infectious Diseases (USAMIID), Fort Detrick, MD, USA

**Keywords:** Crimean–Congo haemorrhagic fever, serology, Bulgaria, livestock

## Abstract

Crimean–Congo haemorrhagic fever (CCHF) is a tick-borne zoonotic disease. Over the past decade, CCHF cases in humans have emerged in Turkey and reemerged in the Balkan countries, Ukraine and Tajikistan. Occupational contact with infected livestock has been recognized as a common cause of the disease. A cross-sectional seroprevalence study in livestock was conducted in farming communities of an endemic area in Bulgaria, southeastern Europe. Overall, 72% of the tested animals were positive for IgG antibodies to CCHF virus. By the time the animals were one-year old almost 50% had serologic evidence of CCHF infection, and by two years already 80% of them had been infected. The data obtained in this study reflect current situation of CCHF virus infection among livestock in Bulgaria. The results showed active CCHF virus circulation that poses risk for humans to be infected during contacts with animals and requires public health awareness.

## Introduction

Crimean-Congo haemorrhagic fever (CCHF) is a tick-borne zoonotic infection. Ticks of the genus *Hyalomma* are the principal vector, and a wide range of animals can become infected. The disease exists over a large geographic area, including Africa, the Middle East, Eastern Europe and Central Asia. In animals (most of the reports involve livestock) the infection is either in-apparent or the animals exhibit minimal symptoms. However, when human cases do occur, haemorrhagic complications and high mortality rates have been described.[1] Over the past decade, CCHF cases in humans have emerged in Turkey and reemerged in the Balkan countries, Ukraine and Tajikistan.[2] This has prompted a renewed interest in this disease. Ever since CCHF was first described, occupational contact with infected livestock has been recognized as a common cause of the disease.[3] Seroprevalence rates in livestock can be as high as 78%.[4] A recent study in Turkey found that 12% of a high-risk human population had serologic evidence of prior CCHF infection.[5] The reason for the apparent resurgence in CCHF activity in endemic foci is unknown, but appears to be part of a general increase in tick-borne disease in Europe and Central Asia.[6] Changes in the environment, land use, hunting, and animal husbandry practices all potentially impact this tick-borne disease.[7]

Human CCHF infections have appeared in previously non-endemic regions in Bulgaria [8] and the European part of Turkey.[9] CCHF was also reported from ticks in the central and the southeast parts of Bulgaria.[10] To provide insight and overview on circulation of CCHF in livestock, a cross-sectional seroprevalence study was undertaken in southeastern Bulgaria.

## Materials and methods

### Study area

This study was conducted in two rural farming communities: Topolitsa and Karageorgievo; both are villages in the municipality of Aytos, about 30 kilometres from District Burgas, in southeastern Bulgaria. This area has shown the highest CCHF seroprevalence rates among humans.[11,12] In the two villages, many residents keep small numbers of livestock. Sheep and cattle were the most common animals kept, with smaller numbers of goats and donkeys. The animals spend part of their time confined to a barn or enclosure, and part of the day grazing outside.

### Sample collection from animals

This study was conducted over three days in September 2011. Blood samples and ticks were collected from sheep, cattle, goats, and donkeys.

Using a pre-printed questionnaire, the following data on each animal were gathered: species, age, sex, country of origin, location of animal, and if any ticks were present on the animals. Once verbal permission was obtained and the questionnaire was filled out, 5–7 ml of blood was drawn from each animal. This work was approved by the NAMRU-3 Institutional Animal Care and Use Committee.

The blood samples were collected from the animals by veterinary personnel. A total of 392 animals were sampled. At the end of each day, the blood was centrifuged at 1300*g* for 10 min and the serum was separated and frozen at −20 °C. When the field work was completed, the frozen samples were transported in liquid nitrogen to the laboratory.

### CCHF–IgG screening test

The IgG ELISAs were performed using a modification of the IgM capture ELISAs that were previously described.[13–15] Briefly, 96-well flat bottom polyvinyl chloride (PVC) microtiter plates (Dynatech Laboratories, Chantilly, Virginia) were coated overnight at 4 ºC with anti-CCHFV hyper-immune mouse ascitic fluid (HMAF) (dilution 1:1000) diluted in 10 mM phosphate-buffered saline (PBS), pH 7.4. The capture plates were washed five times with PBS containing 0.1% Tween 20 (PBS-T) and BPL (betapropiolactone). Following, cobalt irradiated CCHFV-IbAr 10200 strain-infected *Vero E6* supernatant antigen (1:20 diluted in PBS-T containing 5% skim milk (PBS-S, 100 μl/well) was added as a positive antigen control and normal *Vero E6* supernatant was used as negative antigen and incubated for 1 hour at 37 °C. After washing five times with PBS-T, livestock samples, diluted 1:100 in PBS-S, were added and the plates incubated for 1 h at 37 ºC. Samples were tested in duplicate. One known positive control sample and four known negative control samples were included on every assay. After washing, PBS-S diluted horseradish peroxidase-labelled (HRP)-labelled anti-species IgG (KPL Inc., Gaithersburg, MD, USA) was added and the plates were incubated for 1 h at 37 ºC. The plates were washed, ABTS [2,2′-azino-di(-ethylbenzthiazoline-6-sulfonate)] peroxidase substrate (KPL Inc.) was added, and incubated for 30 min at room temperature. The optical densities (ODs) were determined at 410 nm in an automated ELISA reader. Adjusted OD for each sample were determined by subtracting the average OD value of the negative or mock antigen wells from the average OD value of the positive antigen wells. For each assay, the mean and standard deviation of the adjusted OD values was determined for all four negative control samples. The cutoff of each assay was the mean OD value plus three standard deviations rounded up to the nearest tenth. This OD value was typically 0.2. A sample was considered positive if the OD value was greater than or equal to this OD cut-off value.

## Results and discussion

The results from our study showed that, overall, 72% (282/392) of the tested animals were positive for IgG antibodies to CCHFV. Antibodies to CCHFV were found in 71% of the cattle, 74% of the sheep, 50% of donkeys and 60% of goats (Table 1).

**Table 1.** Seroprevalence of CCHF by animal species.

	Cattle	Sheep	Donkey	Goat
Total	127	242	8	15
Number Positive (%)	90 (71%)	179 (74%)	4 (50%)	9 (60%)

By looking at the age distribution of the tested animals, we found that by the time the animals were one year old almost 50% had serologic evidence of CCHF infection, and by three years over 80% had been infected (Table 2).

**Table 2.** Seroprevalence of CCHF by animal age.

	<1 year	1	2	3	4	5–6	7+
Total	21	92	70	47	57	44	48
Number Positive (%)	6 (28%)	44 (48%)	56 (80%)	41 (87%)	45 (79%)	41 (93%)	43 (90%)

The age of 13 animals was unknown.

CCHF incidence and spread increased in recent years. Sporadic cases and even outbreaks are being reported every year. The Balkan Peninsula is well-known endemic region for CCHF.[16–21] Most of Bulgaria is an ecologically favourable environment for CCHF virus circulation in nature. Despite this fact, only a few CCHF cases per year are registered in the last years in Bulgaria: a total of 37 CCHF cases for the period 2007–2012 and case fatality rate is 3/37 (8.1%). Although the number of cases is lower than previously reported, a spread of the disease in new areas is seen [8] and seroprevalence studies have shown rates that vary between 0% and 7.6% in human populations of different localities in Bulgaria.[11,12]

Seroprevalence studies in livestock have not been done in Bulgaria for the last four decades.[22] The results from our pilot study in southeastern Bulgaria (District Burgas) indicated high rates of CCHF sero-positivity among livestock. Establishment of surveillance systems to monitor the viral activity in the region and implementation of vector control plans are highly recommended. All of the animals were born in Bulgaria and none were reported to have been imported. The study documents that the sero-positive rate increased with the animal's age. This finding is consistent with the reported virus prevalence in southeastern Bulgaria.[10]

Despite that the last human CCHF cases in the two villages chosen for our study were recorded about 10 years ago, results of our investigation showed indeed active CCHF virus circulation that poses risk for humans to be infected during contacts with animals and requires public health awareness. Further studies are planned to extend these investigations to animals and ticks in this and other endemic areas in the country to evaluate the risk for humans.

## Conclusions

The obtained data in this study reflect the current situation of CCHF viral infection among livestock in Bulgaria. The results showed active CCHF virus circulation that poses risk for humans to be infected during contacts with animals and requires public health awareness. 
